# Services for people at high risk improve outcomes in patients with first episode psychosis

**DOI:** 10.1111/acps.12480

**Published:** 2015-09-11

**Authors:** P. Fusar‐Poli, C. M. Díaz‐Caneja, R. Patel, L. Valmaggia, M. Byrne, P. Garety, H. Shetty, M. Broadbent, R. Stewart, P. McGuire

**Affiliations:** ^1^ King's College LondonDepartment of Psychosis StudiesInstitute of Psychiatry, Psychology & NeuroscienceLondonUK; ^2^ South London and Maudsley NHS Foundation TrustOASISLondonUK; ^3^ Department of Brain and Behavioural SciencesUniversity of PaviaPaviaItaly; ^4^ School of MedicineChild and Adolescent Psychiatry DepartmentHospital General Universitario Gregorio MarañónIiSGMCIBERSAMUniversidad ComplutenseMadridSpain; ^5^ King's College LondonDepartment of PsychologyInstitute of Psychiatry, Psychology & NeuroscienceLondonUK; ^6^ South London and Maudsley NHS Foundation TrustBiomedical Research Centre NucleusLondonUK; ^7^ King's College LondonDepartment of Psychological MedicineInstitute of Psychiatry, Psychology & NeuroscienceLondonUK

**Keywords:** first episode psychosis, psychosis risk, UHR, ARMS, schizophrenia, prodromal, CRIS, SLaM

## Abstract

**Objective:**

About one‐third of patients referred to services for people at high risk for psychosis may have already developed a first episode of psychosis (FEP). We compared clinical outcomes in FEP patients who presented to either high risk or conventional mental health services.

**Method:**

Retrospective study comparing duration of hospital admission, referral‐to‐diagnosis time, need for compulsory hospital admission and frequency of admission in patients with FEP who initially presented to a high‐risk service (*n* = 164) to patients with FEP who initially presented to conventional mental health services (*n* = 2779). Regression models were performed, controlling for several confounders.

**Results:**

FEP patients who had presented to a high‐risk service spent 17 fewer days in hospital [95% CI: −33.7 to (−0.3)], had a shorter referral‐to‐diagnosis time [B coefficient −74.5 days, 95% CI: −101.9 to −(47.1)], a lower frequency of admission [IRR: 0.49 (95% CI: 0.39–0.61)] and a lower likelihood of compulsory admission [OR: 0.52 (95% CI: 0.34–0.81)] in the 24 months following referral, as compared to FEP patients who were first diagnosed at conventional services.

**Conclusion:**

Services for people at high risk for psychosis are associated with better clinical outcomes in patients who are already psychotic.

**Significant outcomes**

- First episode psychosis (FEP) patients who present to high‐risk services have a lower frequency of hospital admission, fewer days in hospital and a lower likelihood of compulsory admission than FEP patients who present to conventional services.
- These better outcomes may reflect the engagement of patients at an earlier stage of the FEP.
- Services for people at high‐risk may benefit patients who are already psychotic by facilitating earlier detection.

**Limitations**

- Observational study: patients were not randomly assigned to the samples that were compared.
- It was not possible to control for all treatments received from the FEP diagnosis over the follow‐up time.
- However, the use of antipsychotic exposure as proxy index of illness severity and treatment offered after the diagnosis of FEP did not affect the findings.

## Introduction

Over the last two decades, specialised clinical services have been developed for people at high risk for psychosis 1, 2. Providing clinical care at this stage may reduce the risk of these individuals subsequently developing a psychotic disorder 3, 4. However, although they are designed for people who are vulnerable to psychosis, about a third of those referred are found to already be in the first episode of psychosis (FEP) when they are assessed by specialised high‐risk teams 1, 5. This may reflect the fact that the symptoms associated with the high‐risk state are qualitatively similar to those of first episode psychosis, but less severe. As soon as a diagnosis of psychosis is confirmed, high‐risk services immediately refer these patients to specialised first episode services, where specific treatment can be initiated.

In the absence of a specialised service for people at high risk, an individual with signs of high‐risk state would not usually be referred for mental health care: a referral would only be made if the patient was considered to be psychotic. Consequently, a person who was psychotic but incorrectly perceived to be at high risk is likely to be assessed by mental health services sooner when there is a high‐risk team available. Previous studies have highlighted that the longer the delay between the onset of psychosis and the initiation of treatment, the poorer the outcome 6. This suggests that first episode patients who are inadvertently referred to high‐risk services have better clinical outcomes than those whose first contact is with conventional mental health services.

### Aims of the study

This study tested the hypothesis that first episode patients who presented to a high‐risk service had better clinical outcomes compared to those presenting to conventional mental health services, as indexed by number and duration of hospital admissions, referral‐to‐diagnosis time and the proportion of admissions that were compulsory.

## Material and methods

### Data sources

A retrospective review of clinical records was performed using data from the South London and Maudsley NHS Trust (SLaM) electronic Patient Journey System (ePJS), including all information documented by professionals involved in each patient's clinical care. ePJS anonymised clinical data from over 250 000 patients receiving secondary mental health care are available in the SLaM Biomedical Research Council (BRC) Case Register, which facilitates focussed searching and data extraction from structured and unstructured text fields within the electronic health record using the Clinical Record Interactive Search tool (CRIS) 7.

### Samples

#### First episode patients referred to a high‐risk service

These patients were drawn from referrals to OASIS, a clinical service for people at high risk for psychosis in South London 1. On average, approximately one in three of those referred to OASIS meet criteria for a FEP 1. Patients with this diagnosis are assertively referred to an affiliated clinical team specialised in the management of first episode psychosis. OASIS was first implemented in the boroughs of Lambeth and Southwark before later being implemented in the boroughs of Lewisham and Croydon. From January 2001 to September 2011, there were 1090 referrals to OASIS. For data prior to 2007, electronic records of clinical files were not complete, but a scanned copy of the written files was available for manual review.

Between OASIS inception and September 2011, 263 referrals received a diagnosis of psychosis. Of those, 34 received a diagnosis of multiple episode psychosis, whereas two patients had been referred on two occasions to the high‐risk service and were duplicated in the referral log 10 patients initially diagnosed with an at risk state for psychosis (at risk mental state, ARMS) and subsequently reported to have made the transition within 3 months were also included as first episode psychosis, because a retrospective re‐assessment of their symptoms after they were diagnosed with psychosis made the clinicians consider that they were already psychotic when they were referred to the service 5, 8. Thus, the initial first episode sample consisted of 237 patients. Of those, nine cases were not available in the ePJS. After careful clinical file review, 27 patients did not fulfil criteria for a FEP: three patients were actually diagnosed with an at risk mental state and did not make the transition; nine were diagnosed with multiple episode psychosis, and fifteen did not receive a primary diagnosis of psychosis (six patients were diagnosed with affective disorders without psychotic symptoms, four with personality disorders, two with adjustment disorders, one with substance use disorders, one with attention deficit hyperactivity disorder and one with a learning disability). Thus, our sample comprised 201 patients with a confirmed diagnosis of first episode psychosis. Of those, in 37 cases, there was no assessment or assertive intervention by OASIS (four patients did not engage and there were not enough signs of concern to assertively refer them to other specialised services, whereas 33 patients had already passed the psychosis threshold and were screened out before the assessment took place). Thus, the final sample comprised 164 cases with a confirmed diagnosis of first episode psychosis and whose initial management included an active intervention from OASIS (assessment and/or assertive referral to more appropriate services) (see Figure S1).

#### First episode patients who presented to conventional mental health services

In the catchment area served by SLaM, patients thought or identified to have first episode psychosis usually present to generic adult mental health community, home treatment and inpatient teams, or directly to specialised first episode services. The generic mental health teams may subsequently refer these patients to the first episode services, which in SLaM include the Lambeth Early Onset service (LEO), Southwark Team for Early Intervention in Psychosis (STEP), Lewisham Early Intervention Service (Lewisham EIS) and Croydon Outreach Assessment Support Team (COAST). Like OASIS, these first episode services accept self‐referrals and referrals made by health and non‐health agencies 9 and provide a similar form of clinical care which focuses on assertive patient engagement and early clinical intervention 1. In this study, we controlled for potential differences in the provision of treatment by including borough of residence and antipsychotic exposure as covariates in all multivariable analyses.

We compared clinical outcomes of patients whose first contact with mental health services for first episode psychosis was either with a specialised high‐risk service or with conventional services. The conventional services sample (*n* = 2779) was drawn from all patients who presented for the first time between 2007 and 2011 and received a diagnosis of first episode psychosis. The period of 2007–2011 was chosen as 2007 was the first full year in which electronic records were used across all SLaM services. The sample was filtered to exclude any patients who had previously been referred to OASIS with first episode psychosis (in order to ensure that individuals analysed in the high‐risk group were not duplicated in the conventional services group) and to only include patients aged between 14 and 35 at the time of referral to SLaM (to reflect the inclusion criteria of the high‐risk service). Most (about 80%) of this sample (*n* = 2284) presented to generic adult mental health services; a minority presented directly to first episode teams (*n* = 495). Clinical record data for the entire sample were accessed using CRIS, a bespoke software designed to rapidly search electronic records 7.

### Data collection

The following data were extracted from both samples: sociodemographic characteristics (age, gender, ethnicity, marital and employment status, borough of residence), diagnosis, referral‐to‐diagnosis time (measured in days from the date of referral to date of recording of diagnosis), date of first antipsychotic prescription, dates of hospital admissions in a 24‐month follow‐up period, dates of compulsory admissions under the UK Mental Health Act [MHA; which regulates involuntary admission to hospital of people diagnosed with a mental disorder for assessment and/or treatment 10] in a 24‐month follow‐up period and the total cumulative duration of hospital admission in a 24‐month follow‐up period. Ethnicity was recorded according to categories defined by the UK Office for National Statistics 11 and documented in participants’ health records at the time of first presentation to OASIS or conventional mental health services. The initial diagnosis of first episode psychosis was made by the treating clinician according to ICD‐10 criteria 12 and corresponded to the following categories: schizophrenia and related disorders [including patients diagnosed with schizophrenia (F20), delusional disorder (F22) and other schizophrenia‐like disorders (F23, F28 and F29)]; schizoaffective disorder (F25); bipolar disorder [including patients receiving a diagnosis of mania (F30) or bipolar disorder (F31)]; psychotic depression (F32.3, F33.3); drug‐related psychosis (F1x.5); other psychoses. A 24‐month follow‐up period started from the date of referral to OASIS or the conventional service where the diagnosis of first episode psychosis was made. This time period was selected because it permitted assessment of outcomes in the entire sample, and because the outcomes in the first 2 years after illness onset predict the long‐term outcomes 13.

### Statistical analyses

Statistical analyses were conducted to test the association between predictors and outcomes using stata 12 software 14 at a significance level of *P* < 0.05. The main exposure was whether the patients with first episode psychosis had been seen by OASIS or by conventional services. The primary outcome was the cumulative duration of hospital admission (in days) during the 24 months of follow‐up. This was chosen because in patients with psychosis, the duration of admission indicates the degree of disability and is also the greatest contributor to the costs of clinical care 15. Secondary outcomes were the occurrence of compulsory admission to hospital and the frequency of hospital admission between 2 weeks and 24 months of follow‐up. Although it was not possible to investigate differences in the duration of untreated psychosis (DUP), as this variable was not routinely documented in clinical records, an analysis was performed to compare referral‐to‐diagnosis time between patients seen by OASIS or by conventional services as a proxy measure of DUP.

Age, gender, ethnicity, marital status, employment status, borough of residence, diagnosis and exposure to antipsychotics were included as covariates in multivariable analyses. Where missing data were present in covariates, these were included as explanatory variables in multivariable analyses. Further sensitivity analyses were performed including only participants with complete covariate data to assess the potential impact of missing data.

A sensitivity analysis was performed to test whether there were differences between OASIS data collected before and after 2007. Because the conventional services group included first episode services, supplementary three‐way analyses were performed to compare outcomes in patients who presented to the OASIS, first episode services and other SLaM services in order to ascertain any potential differences in outcomes associated with first episode services compared to other SLaM services.

For descriptive analyses, continuous variables were expressed as mean and standard deviation (SD); categorical variables were expressed as frequencies and percentages. Comparison of age distribution between groups was tested using Mann–Witney's *U*‐test for two‐way analyses and anova for three‐way analyses. Chi‐square tests were used to compare groups for discrete categorical variables. No differences in *P* values were found when applying Fisher's exact test to contrasts where individual cell frequency was fewer than five. Owing to non‐proportionality of hazards, multivariable regression methods were employed at varying periods of follow‐up rather than utilising Cox regression for survival analysis. Multiple linear regression models were used to assess the association between engagement by the high‐risk service (vs. conventional mental health services) and number of days spent as an in‐patient in the first 12 months and 24 months after referral to SLaM, and time to diagnosis from referral to the high‐risk service or conventional mental health services. Multivariable binary logistic regression models were used to assess the association of initial management by the high‐risk service (vs. conventional mental health services) with compulsory hospital admission under the UK Mental Health Act at 2 weeks, 1 month, 3 months, 6 months, 12 months and 24 months after referral to services. Multivariable Poisson regression models were used to assess the association of initial management by the high‐risk service or conventional mental health services with the number of admissions at 2 weeks, 1, 3, 6, 12 and 24 months after referral to services. Poisson regression to analyse number of hospital admissions was employed rather than binary logistic regression for any hospital admission to overcome the ceiling effect encountered by the latter method for individuals with multiple hospital admissions during the follow‐up period. Despite a large proportion of zero values for number of hospital admissions, zero‐inflated Poisson models were not meaningfully different to standard models (Vuong *P* > 0.05 for all models). For variables with variance greater than mean, negative binomial regression did not yield meaningfully different results to Poisson models (12 months IRR 0.43; 95% CI: 0.32–0.58, 24 months IRR 0.47; 95% CI: 0.36–0.62). For consistency, standard Poisson regression estimates are therefore presented for all variables.

## Results

### Demographic and diagnostic differences between the samples

The first episode patients that were referred to OASIS were younger and more likely to be male, to belong to an ethnic minority, and to have a schizophrenia spectrum disorder (as opposed to an affective psychosis) than those in the conventional services sample (Table 1).

**Table 1:** Characteristics of patients who were assessed and diagnosed by the high‐risk service or conventional mental health services

	High‐risk service (*n* = 164)	Conventional mental health services (*n* = 2779)	
Mean age (SD)	23.6 (4.88)	25.1 (5.95)	*z* = 3.5 *P* < 0.001
Male gender (%)	112 (68.3%)	1663 (59.8%)	χ^2^ = 4.6 *P* = 0.03
Ethnicity (%)			
Black (Black British/Black			
Caribbean/Black African)	93 (56.7%)	942 (35.6%)	χ^2^ = 30.0
Asian	7 (4.3%)	222 (8.4%)	*P* < 0.001
White	51 (31.1%)	1175 (44.5%)	
Other	13 (7.9%)	304 (11.5%)	
Marital status (%)			
Married/cohabiting	12 (7.5%)	275 (11.0%)	χ^2^ = 2.4
Divorced/separated	5 (3.1%)	99 (4.0%)	*P* = 0.31
Single	144 (89.4%)	2129 (85.1%)	
Employment status (%)			
Employed	36 (22.9%)	145 (19.1%)	χ^2^ = 2.4
Student	31 (19.8%	188 (24.8%)	*P* = 0.31
Unemployed	90 (57.3%)	426 (56.1%)	
Initial diagnosis (%)			
Schizophrenia spectrum	123 (75.0%)	1642 (59.1%)	
Bipolar disorder	8 (4.9%)	142 (5.1%)	
Psychotic depression	6 (3.7%)	312 (11.2%)	χ^2^ = 21.0
Schizoaffective disorder	1 (0.6%)	90 (3.2%)	*P* = 0.001
Drug‐related psychosis	5 (3.1%)	157 (5.7%)	
Other psychosis	21 (12.8%)	436 (15.7%)	
Borough of residence (%)			
Lambeth	111 (67.7%)	473 (17.0%)	
Southwark	40 (24.4%)	472 (17.0%)	χ^2^ = 292.9
Lewisham	11 (6.7%)	442 (15.9%)	*P* < 0.001
Croydon	2 (1.2%)	498 (17.9%)	
Other borough	0 (0.0%)	894 (32.2%)	

SD, standard deviation.

### Primary outcome measure

Multiple linear regression analysis (Table 2) revealed that first episode patients who had been first seen by OASIS spent 17 fewer days in hospital in the 24 months following referral than those first seen by conventional services.

**Table 2:** Primary outcome: association of prior contact with the high‐risk service (*n* = 164) compared to conventional mental health services (*n* = 2779) on number of days spent in hospital

	Cumulative change in number of days spent in hospital B coefficient (95% CI)
12 months	−12.7 (−22.5 to (−2.8))
24 months	−17.0 (−33.7 to (−0.3))

Multiple linear regression adjusted for age, gender, ethnicity, marital status, employment status, diagnosis, borough of residence and whether receiving antipsychotic medication. Follow‐up period commenced from date of referral to the high‐risk service or to conventional mental health services.

### Secondary outcome measures

**Figure 1:**
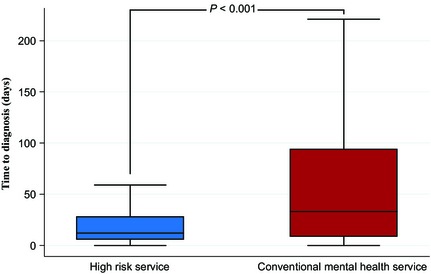
Time to diagnosis in high‐risk service compared to conventional mental health services.

**Figure 2:**
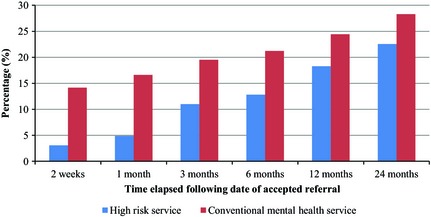
Cumulative percentage of patients detained under Mental Health Act assessed and diagnosed by the high‐risk service (*n* = 164) compared to conventional mental health services (*n* = 2779).

**Figure 3:**
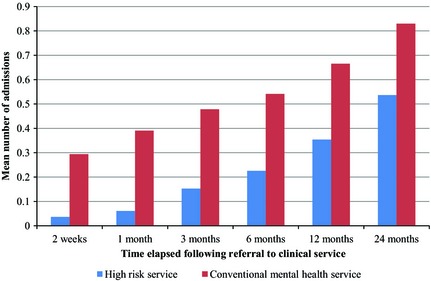
Mean number of hospital admissions following referral to the high‐risk service (*n* = 164) and conventional mental health services (*n* = 2779).

The median referral‐to‐diagnosis time for people with first episode psychosis seen by OASIS was shorter than in those presenting to conventional services (Figure 1). Multiple linear regression analysis comparing OASIS with conventional services corroborated this finding [B coefficient −74.5 days, 95% CI: −101.9–(−47.1)]. Multivariable logistic regression analysis (Table 3) showed that among patients presenting with first episode psychosis, those initially seen by OASIS had a reduced likelihood of compulsory hospital admission in the following 24 months (Figure 2). Multivariable Poisson regression analysis also showed that the patients presenting to OASIS had a lower frequency of admission during the follow‐up period (Figure 3 and Table 3).

**Table 3:** Secondary outcomes: association of prior contact with the high‐risk service (*n* = 164) compared to conventional mental health services (*n* = 2779) on compulsory admission under the UK Mental Health Act and the number of hospital admissions in a given time period

	Any compulsory hospital admission^a^ Odds ratio (95% CI)	Number of hospital admissions^b^ Incidence rate ratio (95% CI)
2 weeks	0.26 (0.10–0.66)	0.13 (0.06–0.30)
1 month	0.29 (0.13–0.62)	0.16 (0.08–0.29)
3 months	0.45 (0.26–0.78)	0.27 (0.18–0.41)
6 months	0.46 (0.28–0.78)	0.34 (0.24–0.48)
12 months	0.53 (0.33–0.84)	0.41 (0.31–0.55)
24 months	0.52 (0.34–0.81)	0.49 (0.39–0.61)

a Multivariable binary logistic regression.

b Multivariable Poisson regression.

All analyses are adjusted for age, gender, ethnicity, marital status, employment status, diagnosis, borough of residence and whether receiving antipsychotic medication. Follow‐up period commenced from date of referral to the high‐risk service or to conventional mental health services.

### Sensitivity and supplementary analyses

A sensitivity analysis comparing the data from OASIS collected between 2001 and 2006 and between 2007 and 2011 (Table S1) did not reveal any significant differences. Sensitivity analyses revealed that there were missing covariate data for ethnicity, marital status and employment status, particularly for the conventional service sample (Table S2). However, multivariable analyses including only participants with full covariate data (Table S3a and S3b) did not significantly differ from analyses including missing data as explanatory variables (Table 2 and Table 3). Supplementary three‐way analyses excluding first episode services (Table S5a/S6a/S7) revealed similar outcomes to the main analyses. A comparison of first episode services with other conventional services showed an association of first episode services with reduced duration of hospital admission (Table S5b) and compulsory hospital admission, a trend towards reduced number of hospital admissions (Table S6b) and no significant difference in referral‐to‐diagnosis time (Table S7). A comparison of OASIS with first episode services revealed a non‐significant trend towards reduced duration of hospital admission (Table S5c), reduced likelihood of compulsory hospital admission and a significant association with reduced number of hospital admissions (Table S6c) and significant reduction in referral‐to‐diagnosis time (Table S7).

## Discussion

To our knowledge, this is the first study to assess the association of presentation to high‐risk services on the outcome of patients referred with a provisional diagnosis of a high‐risk state, but found to have first episode psychosis when assessed by the high‐risk team. We compared clinical outcomes in this group with those in patients with first episode psychosis who presented to generic mental health services, which included specialised first episode teams. We found that in the 2 years following presentation, patients who initially presented to a high‐risk service required fewer hospital admissions were less likely to require compulsory admission and spent fewer days in hospital. These results were independent of differences in age, gender, ethnicity, marital and employment status, borough of residence, psychotic diagnosis and previous exposure to antipsychotic drugs.

The patients who were initially seen by the high‐risk team may have been referred to mental healthcare services earlier than they would have been if a high‐risk team had not been available. The referrers thought (incorrectly) that these individuals were at high risk for psychosis. However, once they had been fully assessed by a specialist team they were found to be already psychotic. This is relatively common among referrals to high‐risk services 1, as it is often difficult to differentiate between the high‐risk state and the early stages of first episode psychosis: the symptoms are qualitatively similar, differing only in severity, and the full clinical picture may not emerge until there has been a detailed and lengthy assessment 16. When there is no high‐risk service, a patient that is perceived as vulnerable but not frankly psychotic may not be referred to mental health services, as these do not conventionally offer clinical support for this group.

The first episode patients who were referred to OASIS may have been more likely to have been mistaken for being in a high risk as opposed to a psychotic state because their clinical presentation did not conform to that typically encountered in first episode patients. In the UK, there is often a long period between the onset of psychosis and first presentation, by which time the patient is acutely disturbed with severe psychotic symptoms. Patients who present at an earlier stage with less overt psychotic symptoms, or whose symptoms had an insidious rather than an acute onset may be more likely to be misclassified as high risk. Further research on the clinical characteristics of this subgroup may help to elucidate whether this is the case. Analysis of the demographic features of the two samples in the present study indicated that the patients initially seen by OASIS were significantly younger than those presenting to conventional services. This is consistent with the notion that these patients may have presented at an earlier stage of the first episode.

We also considered whether the better outcomes in the OASIS sample might reflect the presence of sociodemographic features associated with a relatively good prognosis in‐patients with first episode psychosis, such as female gender 17, not belonging to an ethnic minority 18 or having a non‐schizophrenic psychotic disorder 17. However, comparison of the demographic data from the two samples indicated that the reverse applied: the patients who presented to OASIS were younger, and more likely to be male, from an ethnic minority and to have a schizophreniform psychosis. This may reflect the ethos of high‐risk services like OASIS, which mainly operate in a primary care setting, and are designed to be as accessible to patients and referrers as possible. Referrals can be made from any health or non‐health agency and via self‐referral, and clients are can be seen in their local GP surgery, at home or at a team base in the community. These features may particularly facilitate access to mental health care among patients who are young or who belong to ethnic minority groups 1.

A further potential factor, independent of the nature of clinical features, is that as soon as the patients seen by OASIS had been identified as having first episode psychosis, they were immediately and assertively referred to specialised first episode teams, with an unequivocal diagnosis of first episode psychosis (and not a high‐risk state, or any other diagnosis) that was based on a detailed specialist assessment. This ‘fast‐track’ form of referral with a clear diagnosis to a closely affiliated first episode team may have resulted in a relatively rapid acceptance of the diagnosis by the receiving team (without the need for further assessment), and relatively quicker initiation of antipsychotic treatment. Delays in accessing specialised services for first episode psychosis can significantly increase the interval between the onset of psychosis and the initiation of antipsychotic treatment, the DUP 19, 20. The greater its duration, the greater the duration of hospitalisation and the risk of rehospitalisation during the first 2 years after referral 21.

Although most of the patients who presented to conventional services were seen by generic mental health teams, about 20% of this sample contacted specialised first episode teams directly. We then tested whether the effect of presenting to a high‐risk service was still evident when compared to presenting to a first episode, as opposed to a generic service. Supplementary three‐way analyses showed that the there was still a significant reduction in number of hospital admissions, and in the referral‐to‐diagnosis time in those who presented to the high‐risk service, as well as trends towards reduced duration of hospital admission, and reduced rates of compulsory admission. The persistence of differences relative to patients who presented directly to first episode teams suggests that the beneficial effects of presenting to high‐risk services are not simply a function of being ‘fast‐tracked’ to a specialised first episode care. Rather, it is consistent with the notion that high‐risk services are particularly likely to be referred patients who are in the early stages of the first episode or whose clinical presentation does not immediately suggest that they are psychotic.

In the present study, patients who were initially seen by a high‐risk service had fewer hospital admissions and spent 17 fewer days in hospital within the first 2 years than patients who presented to conventional services. Hospital admissions are the single largest contributor to the direct costs associated with the care of schizophrenia 15. In the UK, the average cost of a night in a psychiatric bed is £350 GBP 15, and an average cost of £12 198 GBP per admission has been estimated 22. We also found that the patients who were initially seen by a high‐risk service were less likely to require a compulsory admission under the Mental Health Act. Compulsory admissions are usually longer than voluntary admissions and are associated with higher direct costs 22. In addition, the experience of compulsory admission can be a negative one for both the patient and their family: this may have an adverse effect on the patient's subsequent engagement with mental health services and their adherence to treatment, and is associated with an increased risk of further compulsory admissions 23.

This was an observational study, and patients were not randomly assigned to the two samples that were compared. However, as high‐risk services are not designed to manage first episode patients, a study in which patients were randomly allocated to high risk and conventional teams would be impractical and ethically problematic. In our study, we investigated variations in clinical outcomes in a single provider of mental health care (SLaM). Another approach which could be investigated in future studies is to compare outcomes in different providers of mental health care depending on whether or not they provide high‐risk clinical services. However, such an approach would not necessarily overcome these limitations because of heterogeneity due to differences in characteristics between different mental healthcare providers. We performed a retrospective assessment of the data and used information that had been entered by clinicians in the patients’ records. Data completion was satisfactory for all the relevant outcomes, and sensitivity analysis did not show significant differences with respect to missing data. However, the information in the clinical records did not include a standardised measure of illness severity at the time of the first episode psychosis diagnosis, and we were therefore unable to control for this potentially confounding factor in the analysis. However, we chose not to employ a propensity score approach as there is evidence that this method does not overcome the limitation of residual confounding 24. We were also unable to control for all treatments received from the first episode psychosis diagnosis over the follow‐up time. However, the use of antipsychotic exposure as proxy index of treatment offered after the first episode psychosis diagnosis did not affect our findings.

This study provides the first evidence that services designed for people at high risk of psychosis may be associated with better outcomes in patients who are already psychotic, but were referred because they were thought to be at high risk. This may result from the referral of patients at a relatively early stage of the first episode and from the fast‐tracking of these patients to specialised first episode services. Both are likely to reduce the interval between the onset of psychosis and the initiation of antipsychotic treatment.

## Acknowledgement

We thank all the OASIS and SLaM patients.

## Declaration of interest

The CRIS team (H.S., M.B., R.S.) have received research funding from Roche; Pfizer; Johnson & Johnson; and Lundbeck. P.M. has received research funding from Janssen; Sunovion; GW Pharmaceuticals; and Roche. Funding organisations had no role in the collection, management, analysis and interpretation of the data; and the preparation, review or approval of the manuscript.

## Ethical approval

Ethical approval for the study was obtained from the Institutional Review Board of the SLaM Psychosis Clinical Academic Group (CAG) for collection and analysis of data on patients presenting to the high‐risk service (OASIS) and Oxfordshire REC C (Ref: 08/H0606/71 + 5) for collection and analysis of data from the BRC Case Register for patients presenting to conventional mental health services.

## Funding

This work was supported by the National Institute for Health Research (NIHR) Biomedical Research Centre at South London and Maudsley NHS Foundation Trust and King's College London; Instituto de Salud Carlos III, Spanish Ministry of Economy and Competitiveness and Fundación Alicia Koplowitz (to C.M.D‐C.); UK Medical Research Council Clinical Research Training Fellowship (MR/K002813/1 to R.P.).

## Supporting information


**Figure S1.** Sample selection for the high risk service.
**Table S1.** Characteristics of patients referred to the high risk service in 2001–2006 compared to patients referred in 2007–2011.
**Table S2.** Characteristics of patients who were assessed and diagnosed by the high risk service or conventional mental health services including missing covariate data.
**Table S3** (a). Primary outcome: association of prior contact with the high risk service (*n = *164) compared to conventional mental health services (*n = *2779) on number of days spent in hospital. Analysis including only participants with full covariate data. (b). Secondary outcomes: association of prior contact with the high risk service (*n = *164) compared to conventional mental health services (*n = *2779) on compulsory admission under the UK Mental Health Act and the number of hospital admissions in a given time period. Analysis including only participants with full covariate data.
**Table S4.** Characteristics of patients who were assessed and diagnosed by the high risk service, first episode service or to other conventional mental health services.
**Table S5** (a) Association of prior contact with the high risk service (*n = *164) compared to other conventional mental health services, not including first episode services (*n = *2284) on number of days spent in hospital. (b) Association of prior contact with the first episode service (*n = *495) compared to other conventional mental health services (*n = *2284) on number of days spent in hospital. (c) Association of prior contact with the high risk service (*n = *164) compared to the first episode service (*n = *495) on number of days spent in hospital.
**Table S6** (a) Association of prior contact with the high risk service (*n = *164) compared to other conventional mental health services, not including first episode services (*n = *2284) on compulsory admission under the UK Mental Health Act and the number of hospital admissions in a given time period. (b) Association of prior contact with the first episode service (*n = *495) compared to other conventional mental health services (*n = *2284) on compulsory admission under the UK Mental Health Act and the number of hospital admissions in a given time period. (c) Association of prior contact with the high risk service (*n = *164) compared to the first episode service (*n = *495) on compulsory admission under the UK Mental Health Act and the number of hospital admissions in a given time period.
**Table S7.** Association of prior contact with the high risk service (*n = *164), first episode service (*n = *495) and other conventional mental health services (*n = *2284) on referral‐to‐diagnosis time from referral to services.
