# Supplementary material for: Services for people at high risk improve outcomes in patients with first episode psychosis
Source: Acta Psychiatr Scand. 2015 Sep 11;133(1):76–85. doi: 10.1111/acps.12480 (PMC4950045; doi:10.1111/acps.12480)
Supplement: Supplementary file 1 — Figure S1. Sample selection for the high risk service. Table S1. Characteristics of patients referred to the high risk service in 2001–2006 compared to patients referred in 2007–2011. Table S2. Characteristics of patients who were assessed and diagnosed by the high risk service or conventional mental health services including missing covariate data. Table S3 (a). Primary outcome: association of prior contact with the high risk service (n = 164) compared to conventional mental health services (n = 2779) on number of days spent in hospital. Analysis including only participants with full covariate data. (b). Secondary outcomes: association of prior contact with the high risk service (n = 164) compared to conventional mental health services (n = 2779) on compulsory admission under the UK Mental Health Act and the number of hospital admissions in a given time period. Analysis including only participants with full covariate data. Table S4. Characteristics of patients who were assessed and diagnosed by the high risk service, first episode service or to other conventional mental health services. Table S5 (a) Association of prior contact with the high risk service (n = 164) compared to other conventional mental health services, not including first episode services (n = 2284) on number of days spent in hospital. (b) Association of prior contact with the first episode service (n = 495) compared to other conventional mental health services (n = 2284) on number of days spent in hospital. (c) Association of prior contact with the high risk service (n = 164) compared to the first episode service (n = 495) on number of days spent in hospital. Table S6 (a) Association of prior contact with the high risk service (n = 164) compared to other conventional mental health services, not including first episode services (n = 2284) on compulsory admission under the UK Mental Health Act and the number of hospital admissions in a given time period. (b) Association of prior contact with the firs [file ACPS-133-76-s001.docx]

**SERVICES FOR PEOPLE AT HIGH RISK IMPROVE OUTCOME IN PATIENTS WITH FIRST EPISODE PSYCHOSIS – SUPPLEMENTARY MATERIAL**

**Figure S1.** Sample selection for the high risk service.

**Referrals to the high risk service (2001-2011)**

**N=1090**

**289 referrals diagnosed with ARMS**

**248 diagnosed with other disorders**

**28 no psychopathology**

**262 not assessed**

**Referrals receiving an initial diagnosis of psychosis**

**N=263**

**34 patients diagnosed with multiple-episode psychosis**

**2 patients repeated in the referral log**

**10 patients initially diagnosed with ARMS making the transition within <3 months**

**Patients with an initial diagnosis of FEP**

**N=237**

**9 not available in the clinical files**

**27 did not fulfill criteria for FEP after careful clinical file review**

**Patients with a confirmed diagnosis of FEP**

**N=201**

**33 found to have passed the threshold and screened out**

**4 did not engage**

**Patients with a confirmed diagnosis of FEP in whom the high risk service played an active role**

**N=164**

Abbreviations: ARMS= At Risk Mental State, FEP= First Episode Psychosis.

**Table S1: Characteristics of patients referred to the high risk service in 2001-2006 compared to patients referred in 2007-2011.**

|  | **High risk service**  **2001-2006**  **(n=72)** | **High risk service**  **2007-2011**  **(n=92)** |  |
| --- | --- | --- | --- |
| Mean age (SD) | 23.7 (5.02) | 23.4 (4.80) | z=0.13  p=0.90 |
| Male gender (%) | 53 (73.6%) | 59 (64.1%) | χ^2^=1.68  p=0.20 |
| **Ethnicity (%)**  Black (Black British/ Black Caribbean/ Black African)  Asian  White  Other | 44 (61.1%)  5 (6.9%)  21 (29.2%)  2 (2.8%) | 49 (53.3%)  2 (2.2%)  30 (32.6%)  11 (12.0%) | χ^2^=7.04  p=0.07 |
| **Marital status (%)**  Married/cohabiting  Divorced/separated  Single | 3 (4.2%)  3 (4.2%)  66 (91.7%) | 9 (10.1%)  2 (2.3%)  78 (87.6%) | χ^2^=2.43  p=0.30 |
| **Employment status (%)**  Employed  Student  Unemployed | 13 (18.6%)  16 (22.9%)  41 (58.6%) | 23 (26.4%)  15 (17.2%)  49 (56.3%) | χ^2^=1.70  p=0.43 |
| **Initial diagnosis (%)**   - Schizophrenia-like - Bipolar disorder - Psychotic depression - Schizoaffective disorder - Drug-related psychosis - Other psychosis | 57 (79.2%)  3 (4.2%)  2 (2.8%)  1 (1.4%)  2 (2.8%)  7 (9.7%) | 66 (71.7%)  5 (5.4%)  4 (4.4%)  0 (0%)  3 (3.3%)  14 (15.2%) | χ^2^=2.96  p=0.71 |

**Table S2.** Characteristics of patients who were assessed and diagnosed by the high risk service or conventional mental health services including missing covariate data.

|  | **High risk service**  **(n=164)** | **Conventional mental healthservices**  **(n=2779)** |  |
| --- | --- | --- | --- |
| Mean age (SD) | 23.6 (4.88) | 25.1 (5.95) | z=3.5  p<0.001 |
| Male gender (%) | 112 (68.3%) | 1663 (59.8%) | χ^2^=4.6  p=0.03 |
| **Ethnicity (%)**  Black (Black British/ Black Caribbean/ Black African)  Asian  White  Other  Not recorded | 93 (56.7%)  7 (4.3%)  51 (31.1%)  13 (7.9%)  0 (0.0%) | 942 (33.9%)  222 (8.0%)  1175 (45.3%)  304 (10.9%)  136 (4.9%) | χ^2^=39.6  p<0.001 |
| **Marital status (%)**  Married/cohabiting  Divorced/separated  Single  Not recorded | 12 (7.3%)  5 (3.1%)  144 (87.8%)  3 (1.8%) | 275 (9.9%)  99 (3.6%)  2129 (76.6%)  276 (9.9%) | χ^2^=14.4  p=0.002 |
| **Employment status (%)**  Employed  Student  Unemployed  Not recorded | 36 (22.0%)  31 (18.9%  90 (54.9%)  7 (4.3%) | 145 (5.2%)  188 (6.8%)  426 (15.3%)  2020 (72.7%) | χ^2^=344.6  p<0.001 |
| **Initial diagnosis (%)**  Schizophrenia-spectrum  Bipolar disorder  Psychotic depression  Schizoaffective disorder  Drug-related psychosis  Other psychosis | 123 (75.0%)  8 (4.9%)  6 (3.7%)  1 (0.6%)  5 (3.1%)  21 (12.8%) | 1642 (59.1%)  142 (5.1%)  312 (11.2%)  90 (3.2%)  157 (5.7%)  436 (15.7%) | χ^2^=21.0  p=0.001 |
| **Borough of residence (%)**  Lambeth  Southwark  Lewisham  Croydon  Other borough | 111 (67.7%)  40 (24.4%)  11 (6.7%)  2 (1.2%)  0 (0.0%) | 473 (17.0%)  472 (17.0%)  442 (15.9%)  498 (17.9%)  894 (32.2%) | χ^2^=292.9  p<0.001 |

**Table S3a: Primary outcome: association of prior contact with the high risk service (n=164) compared to conventional mental health services (n=2779) on number of days spent in hospital. Analysis including only participants with full covariate data.**

|  | Cumulative change in number of days spent in hospital  B coefficient (95% CI) |
| --- | --- |
| 12 months | -15.6 days (95% CI -25.2 to -6.0) |
| 24 months | -22.2 days (95% CI -38.5 to -6.0) |
| *Multiple linear regression adjusted for age, gender, ethnicity, marital status, employment status, diagnosis, borough of residence and whether receiving antipsychotic medication*  *Follow-up period commenced from date of referral to the high risk service or to conventional mental health services* | |

**Table S3b: Secondary outcomes: association of prior contact with the high risk service (n=164) compared to conventional mental health services (n=2779) on compulsory admission under the UK Mental Health Act and the number of hospital admissions in a given time period. Analysis including only participants with full covariate data.**

|  | Any compulsory hospital admission*  Odds ratio (95% CI) | Number of hospital admissions**  Incidence rate ratio (95% CI) |
| --- | --- | --- |
| 2 weeks | 0.16 (0.05 to 0.48) | 0.12 (0.05 to 0.29) |
| 1 month | 0.18 (0.08 to 0.45) | 0.15 (0.08 to 0.30) |
| 3 months | 0.32 (0.17 to 0.61) | 0.29 (0.19 to 0.44) |
| 6 months | 0.33 (0.18 to 0.60) | 0.36 (0.25 to 0.52) |
| 12 months | 0.40 (0.24 to 0.69) | 0.43 (0.32 to 0.58) |
| 24 months | 0.42 (0.26 to 0.70) | 0.48 (0.38 to 0.62) |
| **Multivariable binary logistic regression*  ***Multivariable Poisson regression*  *All analyses are adjusted for age, gender, ethnicity, marital status, employment status, diagnosis, borough of residence and whether receiving antipsychotic medication*  *Follow-up period commenced from date of referral to the high risk service or to conventional mental health services* | | |

**Table S4: Characteristics of patients who were assessed and diagnosed by thehigh risk service, first episode service or to other conventional mental health services.**

|  | **High risk service**  **(n=164)** | **First episode service**  **(n=495)** | **Other conventional mental healthservices**  **(n=2284)** |  |
| --- | --- | --- | --- | --- |
| Mean age (SD) | 23.5 (4.88) | 23.8 (5.16) | 25.35 (6.07) | ANOVA F=19.6  P<0.001 |
| Male gender (%) | 112 (68.3%) | 338 (68.3%) | 1325 (58.0%) | χ^2^=22.5  p<0.001 |
| **Ethnicity (%)**  Black (Black British/ Black Caribbean/ Black African)  Asian  White  Other | 93 (56.7%)  7 (4.3%)  51 (31.1%)  13 (7.9%) | 234 (49.0%)  36 (7.5%)  142 (29.7%)  66 (13.8%) | 708 (32.7%)  186 (8.6%)  1033 (47.7%)  238 (11.0%) | χ^2^=90.3  p<0.001 |
| **Marital status (%)**  Married/cohabiting  Divorced/separated  Single | 12 (7.5%)  5 (3.1%)  144 (89.4%) | 37 (7.7%)  22 (4.6%)  423 (87.8% | 238 (11.8%)  77 (3.8%)  1706 (84.4%) | χ^2^=9.5  p=0.05 |
| **Employment status (%)**  Employed  Student  Unemployed | 36 (22.9%)  31 (19.8%  90 (57.3%) | 35 (21.1%)  33 (19.9%)  98 (59.0%) | 110 (18.6%)  155 (26.1%)  328 (55.3%) | χ^2^=5.2  p=0.26 |
| **Initial diagnosis (%)**  Schizophrenia-spectrum  Bipolar disorder  Psychotic depression  Schizoaffective disorder  Drug-related psychosis  Other psychosis | 123 (75.0%)  8 (4.9%)  6 (3.7%)  1 (0.6%)  5 (3.1%)  21 (12.8%) | 319 (64.4%)  17 (3.4%)  28 (5.7%)  8 (1.6%)  21 (4.2%)  102 (20.6%) | 1323 (57.9%)  125 (5.5%)  284 (12.4%)  822 (3.6%)  136 (6.0%)  334 (14.6%) | χ^2^=61.1  p<0.001 |
| **Borough of residence (%)**  Lambeth  Southwark  Lewisham  Croydon  Other borough | 111 (67.7%)  40 (24.4%)  11 (6.7%)  2 (1.2%)  0 (0.0%) | 171 (34.6%)  107 (21.6%)  73 (14.8%)  75 (15.2%)  69 (13.9%) | 302 (13.2%)  365 (16.0%)  369 (16.2%)  423 (18.5%)  825 (36.1%) | χ^2^=461.7  p<0.001 |

**Table S5a: Association of prior contact with the high risk service (n=164) compared to other conventional mental health services, not including first episode services (n=2284) on number of days spent in hospital.**

|  | High risk service  Cumulative change in number of days spent in hospital  B coefficient (95% CI) |
| --- | --- |
| 12 months | -15.3 (-13.3 to -1.6) |
| 24 months | -20.7 (-37.8 to -3.7) |
| *Multiple linear regression adjusted for age, gender, ethnicity, marital status, employment status, diagnosis, borough of residence and whether receiving antipsychotic medication*  *Follow-up period commenced from date of referral to the high risk service or to other conventional mental health services* | |

**Table S5b: Association of prior contact with the first episode service (n=495) compared to other conventional mental health services (n=2284) on number of days spent in hospital.**

|  | First episode service  Cumulative change in number of days spent in hospital  B coefficient (95% CI) |
| --- | --- |
| 12 months | -7.4 (-13.3 to -1.6) |
| 24 months | -10.5 (-20.5 to -0.6) |
| *Multiple linear regression adjusted for age, gender, ethnicity, marital status, employment status, diagnosis, borough of residence and whether receiving antipsychotic medication*  *Follow-up period commenced from date of referral to the first episode service or to other conventional mental health services* | |

**Table S5c: Association of prior contact with the high risk service (n=164) compared to the first episode service (n=495) on number of days spent in hospital.**

|  | High risk service  Cumulative change in number of days spent in hospital  B coefficient (95% CI) |
| --- | --- |
| 12 months | -7.9 (-18.4 to 2.7) |
| 24 months | -10.2 (-28.1 to 7.7) |
| *Multiple linear regression adjusted for age, gender, ethnicity, marital status, employment status, diagnosis, borough of residence and whether receiving antipsychotic medication*  *Follow-up period commenced from date of referral to the high risk service or to the first episode service* | |

**Table S6a: Association of prior contact with the high risk service (n=164) compared to other conventional mental health services, not including first episode services (n=2284) on compulsory admission under the UK Mental Health Act and the number of hospital admissions in a given time period.**

|  | Any compulsory hospital admission*  Odds ratio (95% CI) | Number of hospital admissions**  Incidence rate ratio (95% CI) |
| --- | --- | --- |
| 2 weeks | 0.20 (0.08 to 0.53) | 0.13 (0.06 to 0.28) |
| 1 month | 0.23 (0.10 to 0.50) | 0.15 (0.08 to 0.28) |
| 3 months | 0.37 (0.21 to 0.65) | 0.26 (0.17 to 0.39) |
| 6 months | 0.39 (0.23 to 0.66) | 0.33 (0.23 to 0.46) |
| 12 months | 0.46 (0.28 to 0.73) | 0.40 (0.30 to 0.53) |
| 24 months | 0.48 (0.31 to 0.75) | 0.47 (0.37 to 0.59) |
| **Multivariable binary logistic regression*  ***Multivariable Poisson regression*  *All analyses are adjusted for age, gender, ethnicity, marital status, employment status, diagnosis, borough of residence and whether receiving antipsychotic medication*  *Follow-up period commenced from date of referral to the high risk service or to other conventional mental health services* | | |

**Table S6b: Association of prior contact with the first episode service (n=495) compared to other conventional mental health services (n=2284) on compulsory admission under the UK Mental Health Act and the number of hospital admissions in a given time period.**

|  | Any compulsory hospital admission*  Odds ratio (95% CI) | Number of hospital admissions**  Incidence rate ratio (95% CI) |
| --- | --- | --- |
| 2 weeks | 0.46 (0.33 to 0.65) | 0.85 (0.70 to 1.02) |
| 1 month | 0.52 (0.39 to 0.70) | 0.84 (0.72 to 0.99) |
| 3 months | 0.61 (0.46 to 0.80) | 0.89 (0.77 to 1.02) |
| 6 months | 0.62 (0.48 to 0.80) | 0.88 (0.77 to 1.01) |
| 12 months | 0.69 (0.54 to 0.88) | 0.91 (0.80 to 1.03) |
| 24 months | 0.81 (0.64 to 1.02) | 0.91 (0.82 to 1.02) |
| **Multivariable binary logistic regression*  ***Multivariable Poisson regression*  *All analyses are adjusted for age, gender, ethnicity, marital status, employment status, diagnosis, borough of residence and whether receiving antipsychotic medication*  *Follow-up period commenced from date of referral to the first episode service or to other conventional mental health services* | | |

**Table S6c: Association of prior contact with the high risk service (n=164) compared to the first episode service (n=495) on compulsory admission under the UK Mental Health Act and the number of hospital admissions in a given time period.**

|  | Any compulsory hospital admission*  Odds ratio (95% CI) | Number of hospital admissions**  Incidence rate ratio (95% CI) |
| --- | --- | --- |
| 2 weeks | 0.44 (0.16 to 1.17) | 0.15 (0.06 to 0.34) |
| 1 month | 0.44 (0.20 to 0.98) | 0.17 (0.09 to 0.33) |
| 3 months | 0.61 (0.34 to 1.09) | 0.29 (0.19 to 0.45) |
| 6 months | 0.62 (0.36 to 1.08) | 0.37 (0.26 to 0.53) |
| 12 months | 0.66 (0.40 to 1.08) | 0.44 (0.33 to 0.59) |
| 24 months | 0.59 (0.38 to 0.94) | 0.51 (0.40 to 0.65) |
| **Multivariable binary logistic regression*  ***Multivariable Poisson regression*  *All analyses are adjusted for age, gender, ethnicity, marital status, employment status, diagnosis, borough of residence and whether receiving antipsychotic medication*  *Follow-up period commenced from date of referral to the high risk service or to the first episode service* | | |

**Table S7: Association of prior contact with the high risk service (n=164), first episode service (n=495) and other conventional mental health services (n=2284) on referral-to-diagnosis time from referral to services.**

|  | Change in referral-to-diagnosis time  B coefficient (95% CI) |
| --- | --- |
| High risk vs. other conventional mental health services | -70.3 days (-98.3 to -42.3) |
| First episode vs. other conventional mental health services | 12.0 days (-4.3 to 28.4) |
| High risk vs.first episode services | -82.3 days (-111.7 to -52.9) |
| *Multiple linear regression adjusted for age, gender, ethnicity, marital status, employment status, diagnosis, borough of residence and whether receiving antipsychotic medication* | |
